# Closed-Loop Hybrid Gaze Brain-Machine Interface Based Robotic Arm Control with Augmented Reality Feedback

**DOI:** 10.3389/fnbot.2017.00060

**Published:** 2017-10-31

**Authors:** Hong Zeng, Yanxin Wang, Changcheng Wu, Aiguo Song, Jia Liu, Peng Ji, Baoguo Xu, Lifeng Zhu, Huijun Li, Pengcheng Wen

**Affiliations:** ^1^School of Instrument Science and Engineering, Southeast University, Nanjing, China; ^2^College of Automation Engineering, Nanjing University of Aeronautics and Astronautics, Nanjing, China; ^3^Jiangsu Collaborative Innovation Center of Atmospheric Environment and Equipment Technology, Nanjing University of Information Sciences and Technology, Nanjing, China; ^4^AVIC Aeronautics Computing Technique Research Institute, Xi'an, China

**Keywords:** brain-machine interface (BMI), eye tracking, hybrid Gaze-BMI, human-robot interaction, augmented reality feedback, closed-loop control

## Abstract

Brain-machine interface (BMI) can be used to control the robotic arm to assist paralysis people for performing activities of daily living. However, it is still a complex task for the BMI users to control the process of objects grasping and lifting with the robotic arm. It is hard to achieve high efficiency and accuracy even after extensive trainings. One important reason is lacking of sufficient feedback information for the user to perform the closed-loop control. In this study, we proposed a method of augmented reality (AR) guiding assistance to provide the enhanced visual feedback to the user for a closed-loop control with a hybrid Gaze-BMI, which combines the electroencephalography (EEG) signals based BMI and the eye tracking for an intuitive and effective control of the robotic arm. Experiments for the objects manipulation tasks while avoiding the obstacle in the workspace are designed to evaluate the performance of our method for controlling the robotic arm. According to the experimental results obtained from eight subjects, the advantages of the proposed closed-loop system (with AR feedback) over the open-loop system (with visual inspection only) have been verified. The number of trigger commands used for controlling the robotic arm to grasp and lift the objects with AR feedback has reduced significantly and the height gaps of the gripper in the lifting process have decreased more than 50% compared to those trials with normal visual inspection only. The results reveal that the hybrid Gaze-BMI user can benefit from the information provided by the AR interface, improving the efficiency and reducing the cognitive load during the grasping and lifting processes.

## Introduction

It has been demonstrated that Brain-machine interface (BMI) can be used for paralysis people to control the robotic arm for the objects manipulation tasks in activities of daily living (Millan et al., 2010). BMI users can directly control the robot using the extracted movement intentions from the brain without any muscular intervention (Schwartz, 2016). Although the user can control the robotic arm in three dimensional space to reach and grasp the objects after training via invasive BMIs (Hochberg et al., 2012; Downey et al., 2016), where the neural activity of the brain is measured using the electrodes placed on the surface of the cerebral cortex or implanted directly into the gray matter of the brain, it is necessary to place the electrodes via surgery procedure with medical risks and fewer patients can benefit from this method (Morgante et al., 2007). Non-invasive techniques, which measure the brain activity from the external surface of the scalp without surgical implantation, are more valuable than the invasive ones, e.g., functional magnetic resonance imaging (fMRI; Gudayol-Ferre et al., 2015), functional near-infrared spectroscopy (fNIRS; Naseer and Hong, 2015), magneto encephalography (MEG; Fukuma et al., 2016), electroencephalography (EEG; Moghimi et al., 2013). The EEG signals acquired by placing the electrodes on the surface of the scalp are mostly studied because of its high time resolution, few risks to the user and requires less expensive equipment.

For the EEG based non-invasive BMI, the EEG signals obtained during visual cue or motor imagery are mapped to the commands for the external devices such as humanoid robots (Duan et al., 2015; Andreu-Perez et al., 2017), virtual helicopter (Doud et al., 2011; Shi et al., 2015), wheelchairs (Kim et al., 2017; Li et al., 2017), locomotion exoskeletons (Lee et al., 2017), telepresence mobile robot (Escolano et al., 2012; Zhao et al., 2017), and even animals (Kim et al., 2016). In order to obtain sufficient number of commands for controlling the robotic arm with multiple degrees of freedom, it is desired to perform the multiple mental states classification (Hortal et al., 2015; Kim et al., 2015; Meng et al., 2016). Nevertheless, it is a challenging task in practice for the BMI user to switch among multiple mental states constantly. In fact, it is much easier for a user to maintain a switch between two mental states than that among multiple states. However, it is unable to provide a sufficient degree of control flexibility in such a way. To overcome this shortcoming, many hybrid methods are proposed by combining BMI with additional signals, such as eye-tracking (Kim et al., 2014), electromyography (Leeb et al., 2011; Bhagat et al., 2016), electrooculography (Ma et al., 2015; Soekadar et al., 2016), fNIRS (Khan and Hong, 2017) and so on, so as to increase the number of commands (Hong and Khan, 2017). Gaze selection is demonstrated to be natural, convenient and faster compared with other interaction approaches (Wang et al., 2016). Therefore, the method has been proposed in Onose et al. (2012) and McMullen et al. (2014) where the target is selected via eye tracking and the classified result of the EEG signals is used to initiate the automatic reaching, grasping and delivering actions by a robotic arm.

Although the hybrid Gaze-BMI system by combing eye-tracking and BMI has shown its ability to help the patients with motor disabilities to complete the sophisticated motor task, recent studies have demonstrated that patients working with assistive devices are not satisfied with fully automatic control by the robot only (Kim et al., 2012; Downey et al., 2016). In other words, it is desired for the BMI user to intervene with the controlling process when working with assistive devices rather than fully automatic control. Nevertheless, it is still a challenging task for the user to control the process of objects grasping and lifting via non-invasive BMI (Popović, 2003). High efficiency and accuracy are hard to achieve, even after extensive training (Lampe et al., 2014). An important reason is that usually only the visual feedback is provided to the BMI user, and the user relies exclusively on the visual feedback during the grasping and lifting processes, which may contribute to a time-consuming and ineffective controlling process (Johansson and Flanagan, 2009; Mussa-Ivaldi et al., 2010). Moreover, studies show that it will cause significant increase of the cognitive load if the user has to rely on the visual inspection only to find out whether the current controlling process is completed (Biddiss and Chau, 2007; Antfolk et al., 2013). Therefore, it is desired by the patients to have more intuitive and understandable feedback approaches in BMI based systems.

To this end, we propose to utilize the AR technique to provide the intuitive and effective feedback for a hybrid Gaze-BMI based robotic arm control system, where the eye tracking system is used for the robot position control (i.e., the target selection) and the movement intention is decoded from the EEG signals as the confirmation of the target position selected by the user or the trigger command to be executed on the target. Experiments for the objects manipulation tasks while avoiding the obstacle in the middle of the workspace are designed, where the manipulation tasks are divided into five phrases: reaching, grasping, lifting, delivering and releasing. For the grasping and lifting tasks that requires fine operations, the human supervisory is often desired. For the less demanding tasks, i.e., reaching, delivering and releasing, they can be automatically completed by the robotic arm once the movement intention is detected from the EEG signals. Therefore, our main idea is to maintain as much manual control as possible in the grasping and lifting processes using the hybrid Gaze-BMI, while providing the user with the enriched visual information about the gripper status through the AR technique in real time. The performance of the hybrid Gaze-BMI based systems both in open-loop (with visual inspection only, without AR feedback) and close-loop (with AR feedback) will be compared in the experiments.

The rest of the paper is organized as follows: section Materials and Methods describes the components of the proposed system as well as the experimental protocols used in this study. The results of the experiments are presented in section Results. The discussion of this study is provided in section Discussion and followed by the conclusion in section Conclusion.

## Materials and methods

### System architecture

**Figure 1:**
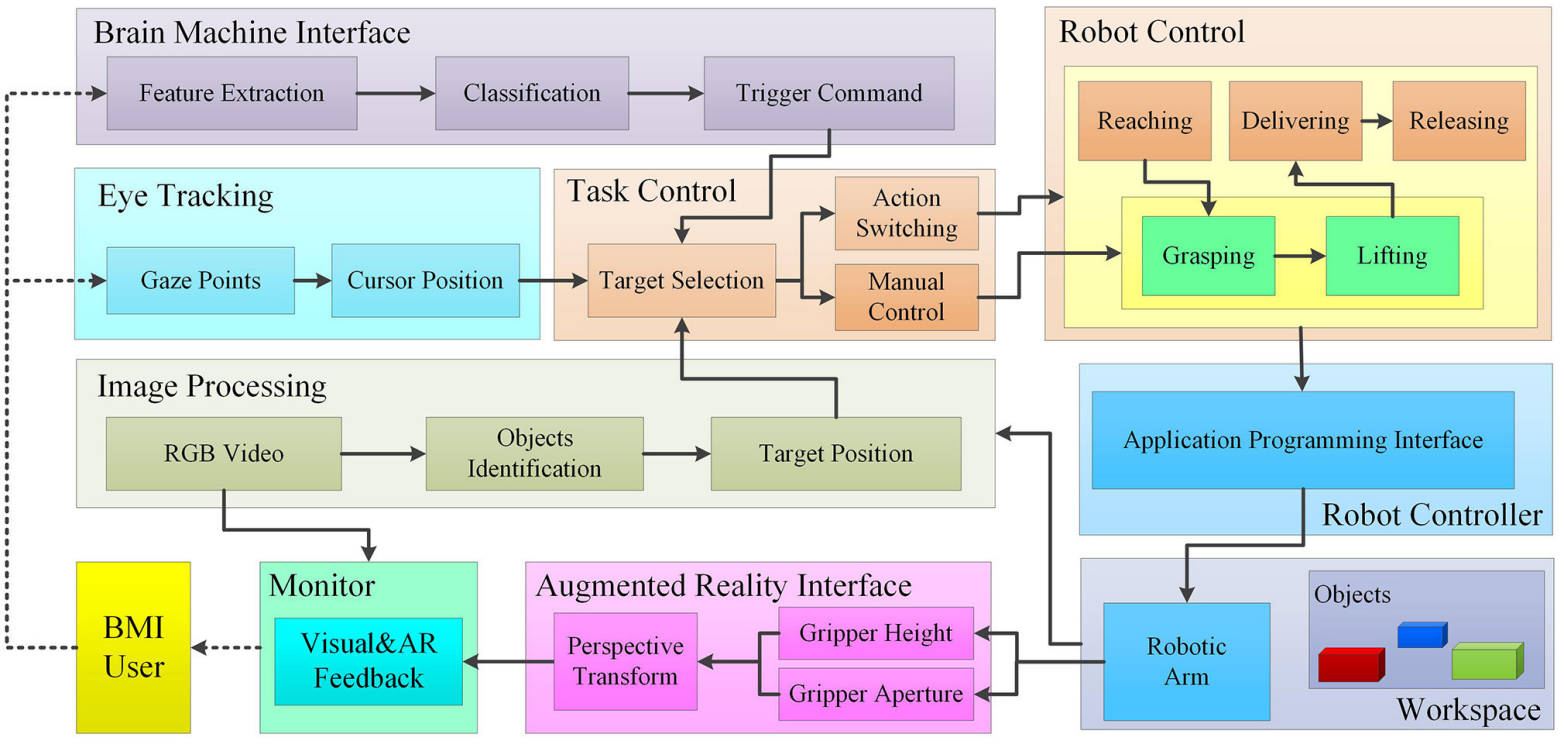
The block diagram of the proposed hybrid Gaze-BMI based robotic arm control system with AR feedback. Image processing is applied to segment all the potential cuboids from the image of the workspace. The segmented objects can be selected by the subjects via eye tacking and confirmed using the trigger commands from the BMI. The initiation commands from the hybrid Gaze-BMI are used to (1) confirm the object selection by the user, or (2) trigger the switching of action sequence or (3) constantly control the aperture and height of the gripper during the grasping and lifting processes, respectively. AR feedback is provided to the BMI user during the grasping and lifting processes via the monitor. The robotic arm implements the reaching, grasping, lifting, delivering and releasing tasks, in response to the trigger commands obtained from the hybrid Gaze-BMI.

**Figure 2:**
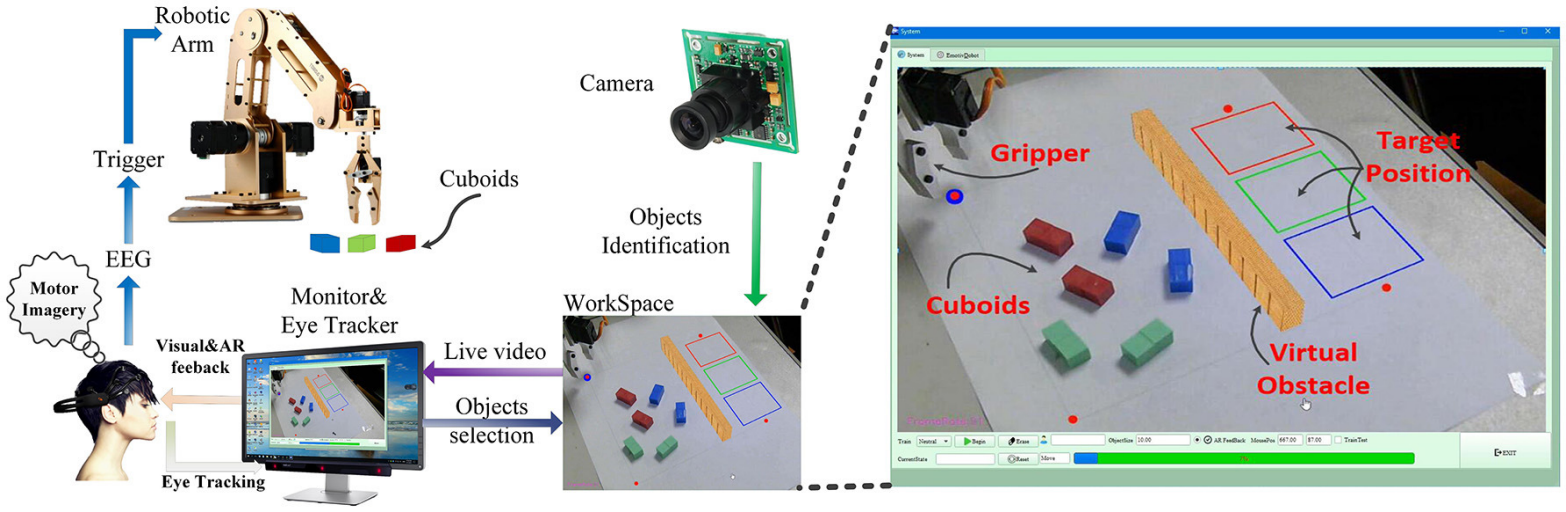
Experimental setup used in this study. The live video of the workspace captured by the camera and the enhanced visual feedback are presented to the user via the monitor. Using the eye-tracking device EyeX, the user can select the object that he/she intends to manipulate. The movement intention can be detected by the BMI device Emotiv EPOC+, which can confirm the user's selection or initiate the control on the selected object. Dobot executes the reaching, grasping, lifting, delivering, and releasing tasks in response to the trigger commands from the user. The enlarged Graphical User Interface, which is programmed in C++ under Qt framework, is shown on the right side of the picture above.

The block diagram of the proposed system is shown in Figure 1. The functional modules of BMI, eye tracking, image processing, automatic control and AR interface are integrated in this system to allow the user performing the objects manipulation tasks. Image processing is applied to segment all the potential cuboids from the image of the workspace. The segmented objects can be selected by the subjects via eye tracking. The outputs decoded from the BMI are used to (1) confirm the object selection by the user, or (2) trigger the switching of action sequence, or (3) constantly control the aperture and height of the gripper during the grasping and lifting processes, respectively. The intentionally selected object by the user as well as the status of the grasping and lifting operations is visually fed back to the user via the computer screen using AR techniques in real time. Eventually, the robotic arm implements the reaching, grasping, lifting, delivering and releasing tasks, in response to the outputs decoded from the hybrid Gaze-BMI. The experimental setup used in this study is shown in Figure 2. The physical system is composed of an eye tracker, an EEG headset, a PC, a robotic arm, and an USB camera. The participants are seated in front of the computer comfortably wearing the EEG headset on their head to perform the object manipulation tasks. The distance from the user to the “23.6” LCD monitor is ~90 cm. The monitor displays the live video captured from the workspace. The interaction between the subjects and the system is via the hybrid Gaze-BMI and the enhanced visual feedback by AR.

### Brain-machine interface

A low-cost commercial EEG acquisition headset, Emotiv EPOC+ (Emotiv Systems Inc., USA), is used to obtain the user's intention to rest or to perform hand motor imagery. This device is consisted of 14 EEG channels (AF3, F7, F3, FC5, T7, P7, O1, O2, P8, T8, FC6, F4, F8, and AF4) and two reference channels (P3, P4). The data are sent to the computer through Bluetooth with a sampling rate of 128 Hz.

**Figure 3:**
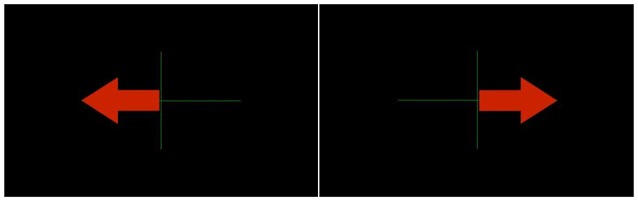
The graz motor imagery BCI stimulation in openvibe. The right arrow and left arrow are used to guide the user to perform the motor imagery and the relax task, respectively.

The OpenVibe toolbox is used for the training session of the BMI decoding model. Firstly, the Graz Motor Imagery BCI Stimulation in the OpenVibe toolbox is used as the EEG signals acquisition paradigm, where the right arrow and the left arrow are shown in a random order to guide the user for the motor imagery tasks as is shown in Figure 3. When the right arrow is presented, the user should imagine the right hand movements until the green cross in the window disappears, while the user should keep relaxed when the left arrow or no arrow is presented. Participants are asked to remain relaxed to reduce the effects from muscle signals during the EEG recording process. Nextly, the pre-processing and feature extraction are applied on the EEG data. A 5th-order Butterworth band pass filter is utilized for temporal filtration with cut-off frequency from 8 to 12 Hz. The filtered signals are then segmented with a 1s-long sliding window in steps of 62.5 ms. The commonly used feature extraction method, i.e., common spatial pattern (CSP), is applied on the signals to extract the features that discriminates between the hand motor imagery and the relax states. Subsequently, a linear discriminant analysis (LDA) classifier is trained to classify the two mental states. Finally, the learned CSP filter and the LDA classifier are applied for the online user intent identification. Two kinds of brain states, i.e., rest and motor imagery, are classified from the EEG signals, alone with an action power, a unidimensional scalar index ranging between 0 and 1 representing the detection certainty that the user has entered the “motor imagery” state. To achieve a reasonable trade-off between true positives and false positives, the detection certainty threshold for the “motor imagery” state is set to 0.60 by rule of thumb in our experiments. Namely, motor imagery state with the detection certainty above 0.60 is used to initiate the execution of a command, otherwise the decoded mental state will be deemed as the “rest” state. The movement intention decoded by OpenVibe is delivered to the robotic arm control engine through the Analog VRPN Server in the OpenVibe every 62.5 ms. When the robotic arm is in operation, no action will be executed.

### Image processing and eye tracking

An USB camera, with a resolution of 1,280 × 720 pixels, is used to capture the live video data of the workspace and sends the video to the computer via an USB 2.0 connection. For the eye tracking, a commercial desktop eye tracker, EyeX (Tobii AB Inc., Sweden), is used to detect and map the user's pupil position to the cursor on the monitor. The eye tracker is fixed at the bottom of the computer monitor (cf. Figure 2). The data are transmitted to the computer via USB 3.0 at a rate of 60 Hz. The gaze points acquired from the EyeX system are filtered to remove the minor gaze fluctuations, which is achieved by calculating the 10-point moving average. Then the filtered gaze points are fed to the computer for updating the position of the cursor position on the monitor every 30 ms.

Image processing and eye-tracking are used for the objects identification and selection in the manipulation tasks. Three kinds of cuboids (10 × 20 × 10 mm) with different colors (red, green and blue) are used in the experiment (cf. Figure 2 right). Cuboids in the workspace are detected using image processing techniques based on their colors. Firstly, the image of the workspace is converted from the RGB space to the HSV space to lessen the illumination effect from the natural environment. Subsequently, the contours of the objects in the image are confirmed based on the threshold of different colors. Finally, all the potential cuboids are segmented from the image of the workspace. It is necessary to perform the calibration procedure for the eye tracker before the experiment. The calibration procedure lasts <1 min for each subject, during which the user gazes at seven points shown on the computer monitor one by one.

The user can move the cursor on the monitor over the target to be manipulated, and then a visual feedback is provided to the user by highlighting a red rectangle surrounding the target (cf. **Figure 7A**). When the object is confirmed by the subject, i.e., when the subject fixates upon the object and the motor imagery state is detected from the EEG signals, the color of the rectangle changes from red to green (cf. **Figure 7B**). Similarly, the switch of the action sequence will be triggered when the user fixates their gaze points on the specific position and meanwhile the movement intention is detected. For example, when the target position for placing the objects is fixated on with the motor imagery state being decoded from the EEG signals, the action sequence will switch from the lifting process to the delivering process.

### Robotic arm

For the actuated system, a desktop robotic arm with 5° of freedom, Dobot (Shenzhen Yuejiang Technology Co Inc., China), is used. The robotic arm controller can directly convert the XYZ position to the corresponding joints positions based on the inverse kinematics. Therefore, the user can directly give the motion end-point information in 3D environment via the hybrid Gaze-BMI, and the controller of Dobot will plan the path to the target position automatically. Then the robotic arm executes the manipulation tasks in response to the trigger commands from the hybrid Gaze-BMI user.

**Figure 4:**
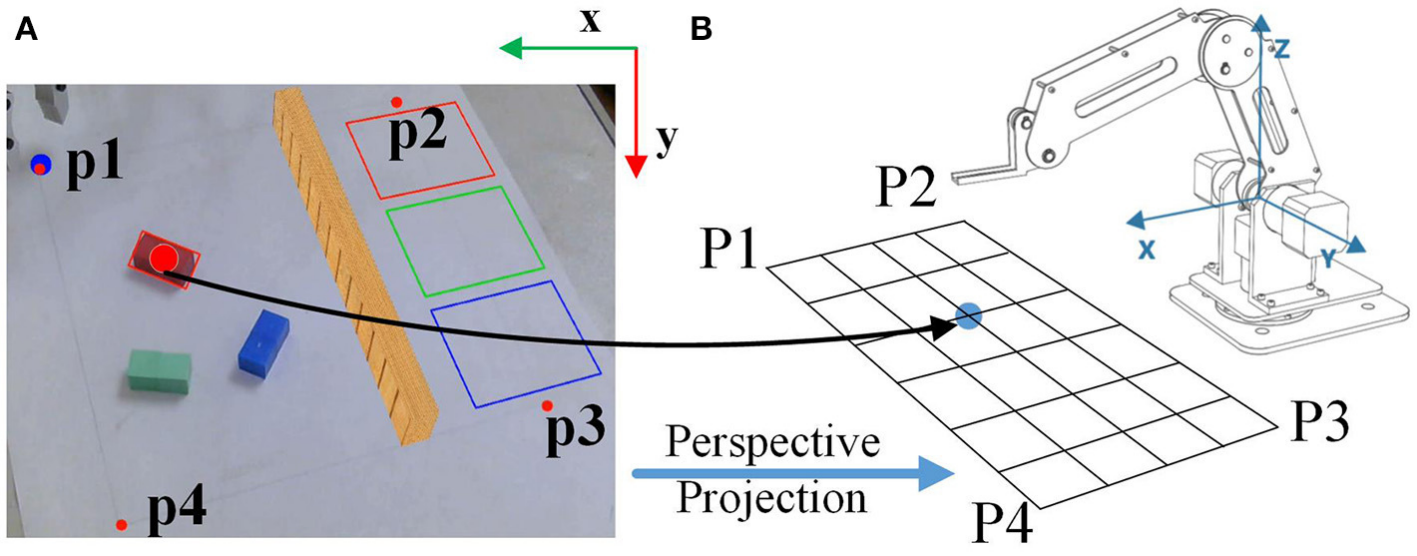
Mapping of the object coordinates from the image panel to those of the robotic arm workspace. **(A)** The coordinates of the object in the image panel. **(B)** The coordinates of the object in the robotic arm workspace.

The workspace is predefined using a rectangle (150 mm × 150 mm) in the real scene. The webcam screen view coordinates will then be mapped with the corresponding robot workspace coordinates, as is shown in Figure 4. Firstly, the coordinates of the vertexes (p1, p2, p3, and p4 in Figure 4A) in the image plane are acquired. Nextly, the pose value of the robotic arm in the four vertexes (P1, P2, P3, and P4 in Figure 4B) of the rectangle is obtained. Subsequently, a perspective transform matrix from the pixels to the coordination of the robotic arm is calculated based on the calibration data (p1~p4 and P1~P4). Finally, the position of the objects in the image plane of the workspace is mapped to the coordination of the robotic arm based on the perspective projection. The commands are sent to the robotic arm engine via its Application Programming Interface (API). In this way, the height and the aperture of the gripper can be obtained from the Dobot engine in real time, so as to present the current state of the tasks to the user with the AR feedback.

### Augmented reality interface

**Figure 5:**
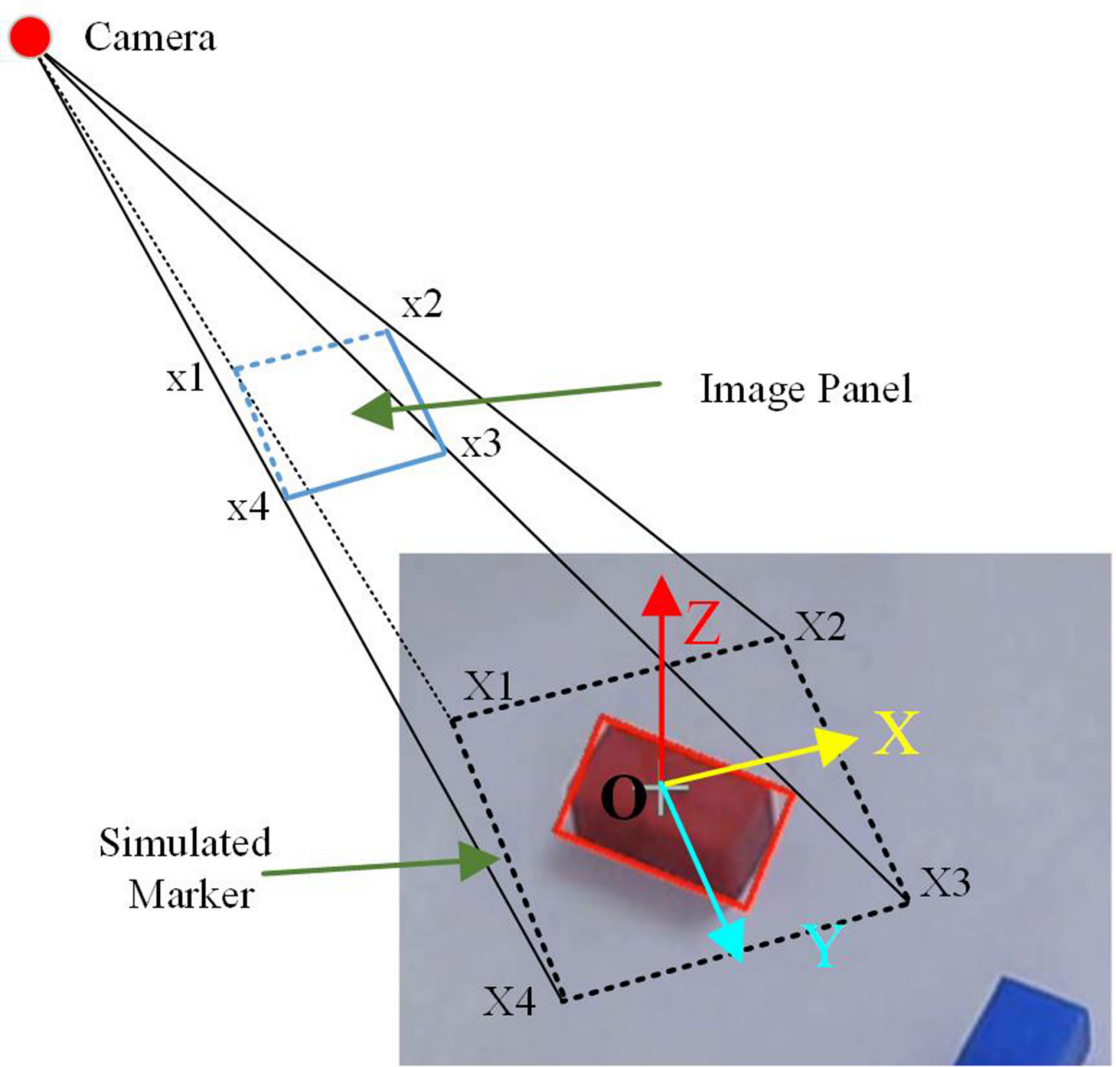
The maker-based tracking method to calculate the camera pose relative to the real world.

The AR interface is implemented with OpenCV and OpenGL. The marker-based tracking method is used to calculate the camera pose relative to the real world to align the real camera and the virtual camera in OpenGL. Firstly, the camera is calibrated using a chessboard. The distortion parameters and the intrinsic parameters of the camera are obtained during the calibration procedure. Then, the extrinsic parameters should be solved, which encode the position and the rotation of the camera relative to the 3D world. To calculate the extrinsic parameters, a square with the same center of the cuboids is used as the simulated marker, as shown in Figure 5. The width of the square is 1 mm, which is calibrated in advance. The virtual objects are of the same size of the virtual markers. Therefore, the size of the virtual objects can be controlled by the size of the simulated marker. The center of the square (O) is assumed to be (0, 0, 0) in 3D world. Then the extrinsic parameters can be solved using solvePnP in OpenCV (Opencv, 2017). Finally, a perspective projection in OpenGL with the field of view and the aperture angle of the camera from intrinsic parameter are obtained, and the virtual camera in OpenGL is put in the position given by the extrinsic parameters to align the virtual and real objects.

In the objects manipulation tasks, the AR feedback is provided to the user during the grasping and lifting processes. Firstly, the enriched visual information, such as the virtual gripper aperture and the simulated grasping force, is presented to the user on the screen during the grasping process in real time. A virtual box whose length is of the same with the aperture of the gripper is placed near to the object, representing the information about the gripper aperture (**Figure 7C**). When the gripper aperture becomes smaller than the width of the object, i.e., the objects has been grasped by the gripper, the grasping force then will be simulated by two arrows normal to the gripper that are overlaid over the cuboid in the image (**Figure 7D**). In addition, the greater the difference between the size of the object and the aperture of the gripper is, the longer the arrows are (i.e., the stronger the grasping force is). Secondly, during the lifting process, the altitude of the gripper is fed back to the subject through the height of the virtual box in the middle of the virtual obstacle (see **Figure 7F**). The altitude of the gripper on the table is calibrated in advance. The height of the virtual box is calculated by the difference value between the real time pose data of the robotic arm in vertical direction and the height of the gripper on the table.

### Experimental protocol

Experiments for the objects manipulation tasks are designed to evaluate whether the hybrid Gaze-BMI users can benefit from the AR feedback for the grasping and lifting processes, where the human supervisory is involved. The workspace is shown in the right side of Figure 2. The user is instructed to select and grasp the object, then deliver it to the target position. The height of the virtual obstacle is 15 mm, which should be avoided by the robotic arm during the delivering process. The object should be released to the rectangular area with the same color as the object. The grasping and lifting processes are controlled manually by the BMI user, i.e., the user will decide when to stop the grasping process and whether the height of the gripper is enough for a safe delivering. The complete objects manipulation protocol is introduced as follows.

#### Reaching

Several cuboids in different colors are placed randomly in the workspace with different orientations (Figure 2 right). The cuboid will be highlighted with a virtual red rectangle surrounding it when the cursor (gaze point) is over it (**Figure 7A**). Once the reaching action is triggered successfully, i.e., the gaze point is being over the object and the motor imagery state has been decoded from the BMI, the color of the rectangle surrounding it will change from red to green indicating the confirmation of the selected object (**Figure 7B**). The position of the selected object in the workspace is mapped to the coordination of the robotic arm as the end-point information. Then the robotic arm will move to the pre-grasp position over the objects. The orientation of the gripper will be adjusted automatically, according to the angle of the object in the workspace based on the image processing results. If a motor imagery state is detected from the EEG signal while no object is being selected, this command will be ignored by the system.

#### Grasping

**Figure 6:**
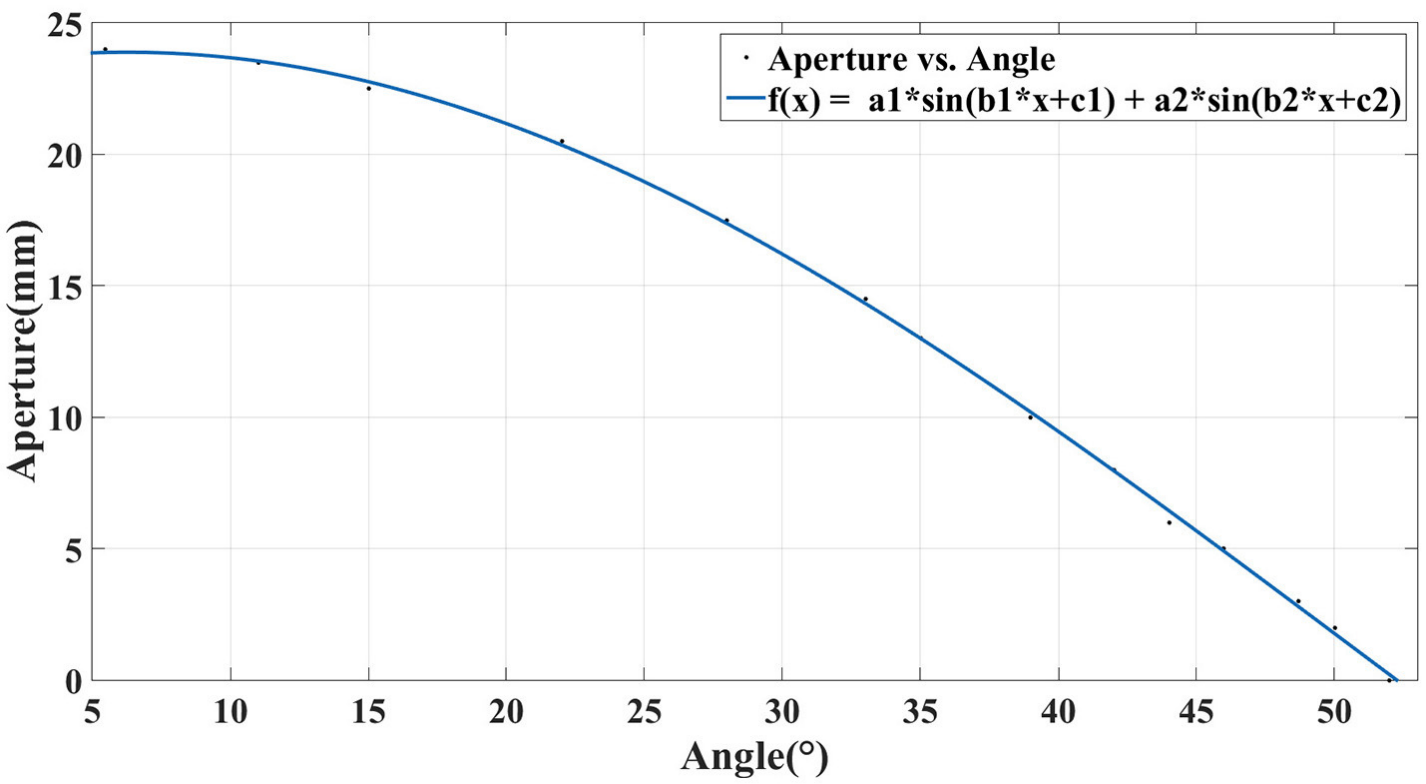
The relation between the aperture of robotic gripper and the angle of the servo.

**Figure 7:**
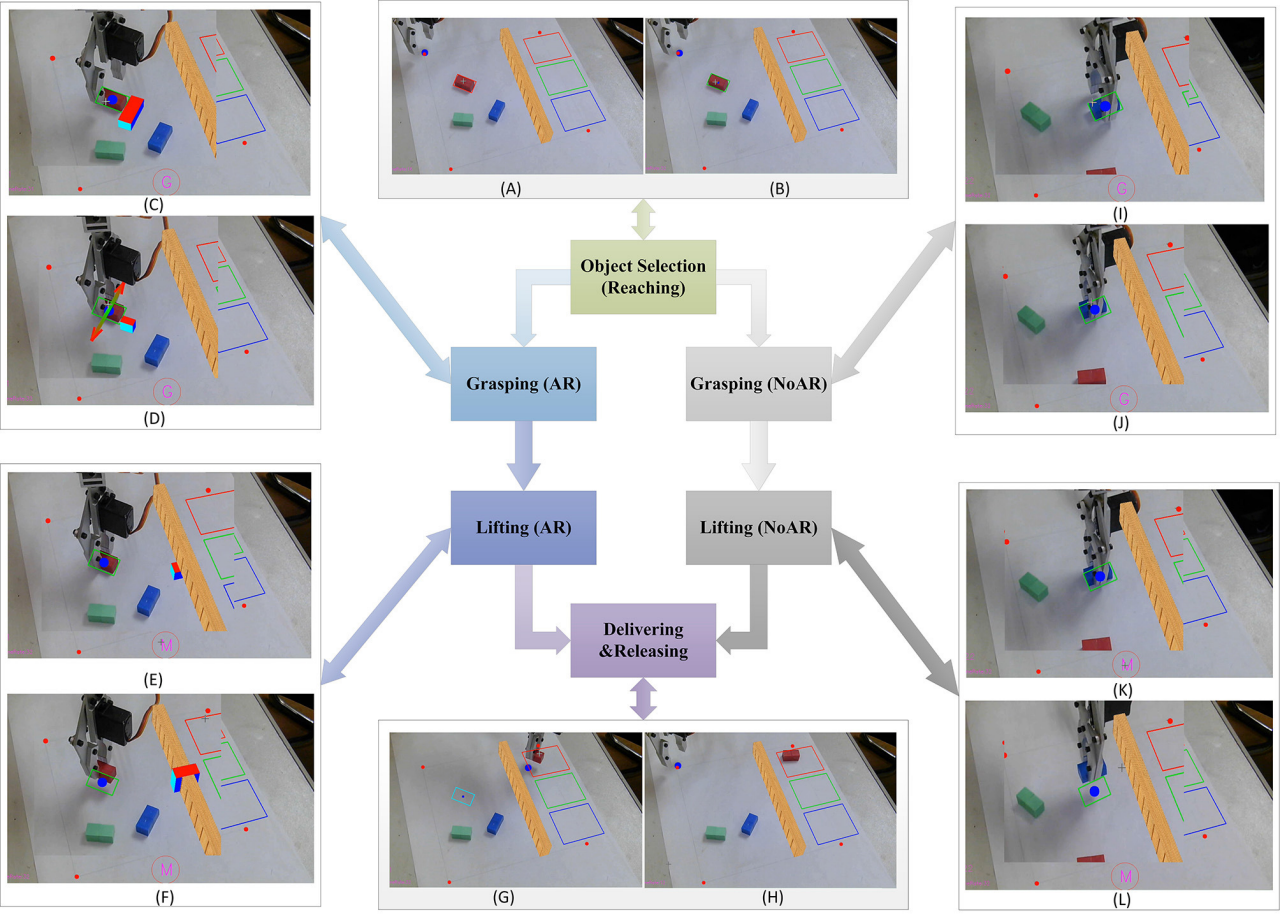
The process of objects manipulation tasks with and without AR feedback. The area of the gripper is expanded as is shown in **(C–L)**. Reaching: **(A)** The robotic arm is in the initial position. An object can be selected by the gaze points of the user, and a red rectangle will then appear around the object, indicating that the user is starring at it. **(B)** The color of the rectangle changes from red to green when the target object is confirmed by the user once the motor imagery state is detected. Next, the robotic arm moves to the position for the subsequent grasping. Grasping (AR): **(C)** The circle with a letter “G” in it will appear at the bottom of the GUI, indicating that the user has arrived at the grasping phrase. The orientation of the gripper is adjusted automatically based on the orientation of the object in the workspace. The aperture of the gripper is presented to the user based on AR feedback interface via a virtual box near the object. **(D)** When the selected object has been grabbed tidily, two virtual arrows normal to the gripper are then overlaid over the object, simulating the grasping force. Lifting (AR): **(E)** the letter in the circle changes from “G” to “M” indicating a successful switching of action sequence from the grasping process to the lifting process. The user can control the gripper moving in the vertical direction to lift the object. The height of the gripper to the table is represented by that of a virtual box in the middle of the obstacle. **(F)** When the height of the virtual box is higher than the obstacle, it is deemed that the altitude of the robotic arm is enough for a save delivering. Delivering and Releasing: **(G)** when the lifting process is completed, the user fixates his/her gaze on the target rectangle and performs motor imagery to trigger the robotic arm moving to the target position automatically. Besides, the color of the rectangle around the object changes from green to cyan, indicating a successful action sequence switching. **(H)** The object is released in the target position. Then Dobot returns to the initial position automatically, waiting for the next trial. Grasping and Lifting (NoAR): **(I–L)** the grasping and lifting processes without AR feedback, where the hybrid Gaze-BMI user has to decide when to stop the current process by the visual inspection only.

Subsequently, the aperture of the gripper will be controlled manually by the user. The gripper is open in the initial state with an aperture of 25 mm. The aperture of the gripper will decrease 1 mm each step in the grasping process if the user maintains the motor imagery state and meanwhile fixates on the object in the image panel. The aperture of the gripper is mapped to the angle of the servo to accomplish the control of the gripper. The relation between the aperture of the robotic arm and the angle of the servo is estimated based on data fitting, as is shown in Figure 6. The circle with a letter “G” in it will appear at the bottom of the GUI, indicating that the user has arrived at the grasping phrase. The width of the virtual box changes with the aperture of the gripper (Figure 7C). The arrows shown in the video means that the grasping force is being generated on the object (Figure 7D). If the cuboid has already been grasped tidily while the user insists on generating the trigger commands, the gripper will continue responding to the commands, and the length of the arrow will continue to increase so as to present the increasing of the grasping force.

#### Lifting

Then the individual should switch the grasping process to the next action sequence that picking the object up to avoid the obstacle. The user should fixate their gaze at the red circle with a letter “G” inside at the bottom of the GUI and perform motor imagery to initiate the switch. The letter in the red circle changes from “G” to “M” indicating a successful state switching from the gasping process to the lifting process (Figure 7E). After that the user is able to control the robotic arm by moving in the vertical direction to avoid the virtual obstacle in the middle of the workspace. The height of the robotic arm will increase 1 mm in response to each trigger command from the hybrid Gaze-BMI. A virtual box, whose height is equal to the altitude of the robotic gripper obtained from the Dobot engine, will be presented right in the middle of the virtual obstacle. In this way, the subject can easily find out whether the height of the robotic gripper is enough for a safe delivering (Figure 7F).

#### Delivering and releasing

Subsequently, the subject may switch from the lifting process to the delivering process, by fixating his gaze to one of the three target rectangular areas in different colors and then performing motor imagery. Then the Dobot will generate a path in the plane with the same height as that of the gripper and deliver the object to the target position automatically (Figure 7G). Finally, once the OpenVibe has detected the motor imagery state from the EEG signals, the object will be released and the robotic arm returns to the initial position automatically, waiting for the next trial (Figure 7H).

Grasping and lifting processes in open-loop (with visual inspection only, without AR feedback) are also implemented for the comparison with the same protocol above. Figure 7 shows the whole process in the object manipulation tasks both with and without AR feedback. In the open-loop protocol, the user decides when to stop the grasping and lifting processes by visual inspection only, as is shown in Figures 7I–L.

### Performance evaluation

Eight participants (all males, 24.5 ± 1.2 years old) are recruited from the campus to perform the objects manipulation tasks using the proposed system. All of them are healthy and right handed. This study is carried out in accordance with the recommendations of the Ethics Committee of Southeast University with written informed consent from all subjects. All subjects gave written informed consent in accordance with the Declaration of Helsinki.

Firstly, the BMI decoding model was trained for each subject in the training session described in the subsection brain machine interface. The training session for each subject was composed of a randomly sorted sequence of 40 trials, 20 for the hand motor imagery tasks and 20 for the relax tasks. The execution of each task lasted for 4 s, and it was spaced from the beginning of the next task with an interval lasting randomly from 1 to 3 s, during which the subject could relax concentration. Each task was triggered through visual cues displayed on the screen. The 5-fold cross-validation BMI decoding performance on the data from the training session is then reported.

Secondly, the online evaluation of the robotic arm control system based on the hybrid Gaze-BMI with or without AR feedback was performed. For each subject, the online evaluation session consisted of a randomly sorted sequence of 30 trials, 15 for the system with AR feedback and 15 for the system without AR feedback (i.e., with normal visual inspection only). The online decoding model of BMI is obtained by training with all the data from the training session above. For each online trial, the BMI user operates the robotic arm to transfer a cuboid to the target area in the same color while avoiding the virtual obstacle in the middle of the workspace. The subject can have a rest whenever needed between two trials. We do not limit the task completion time for each trial and the user is asked to bare successful grasping and safe delivering in mind. Therefore, all the subjects can successfully accomplish the object transferring task both with and without the AR feedback.

The online manipulation performance will be evaluated with the following two indices: (1) The number of trigger commands used in both the grasping and the lifting process, as used in Tonin et al. (2010) and Kim et al. (2012). The BMI user generates the trigger commands with the hybrid Gaze-BMI, thereby the number of commands used in the grasping and lifting processes can be used to characterize the efforts of the hybrid Gaze-BMI users with or without AR feedback during the object manipulation tasks. When the object has already been grasped tidily while the user still maintains the motor imagery state and fixates on the object, the robotic arm will continue to execute the trigger commands. Though the aperture of the gripper may not change dramatically, the contact force on the object will increase which may be harmful to the object and the robotic gripper. Similarly, when the height of the gripper is enough for a safe delivering while the user still produces the trigger commands, the gripper will continue moving in the vertical direction. Those unnecessary mental commands will increase the workload of the BMI users and reduce the efficiency of the controlling process. (2) The height gap of the robotic gripper in the lifting process. This index is used for the following considerations. When the BMI user move their gaze point to the target area and perform motor imagery to finish the lifting process, the robotic arm will move to the target area in the plane with the same height as that of the gripper. An ideal condition is that the final height of the robotic gripper in the vertical direction (Z) is just fine for a safe delivering over the obstacle. Therefore, the height gap of the gripper in the lifting process is defined as the altitude difference between the gripper and the obstacle. Those two indices are used to evaluate whether the BMI user can benefit from the AR feedback to successfully complete the delivering task with less efforts. The performance difference between the proposed approach with AR feedback and the one with visual inspection only was evaluated using the one-tailed Wilcoxon rank sum test.

## Results

### The classification performance of the BMI

The 5-fold cross-validation classification accuracy of the BMI for each subject is shown in Table 1. The average classification accuracy for the relax state is 85.0 ± 6.3%. An average accuracy of 86.4 ± 6.4% for the motor imagery state is achieved using the BMI decoding model. The aggregated classification accuracy across the subject is 85.16%, with a standard deviation of 4.83%. The highest accuracy of the BMI achieved on subject 6 is 94.01%. Subject 7 has obtained the worst performance with an average accuracy of 77.42%.

**Table 1:** The BMI cross-validation classification accuracy for each subject.

**Participant ID**	**Correct rate (%)**		
	**Relax**	**Motor imagery**	**Total**
S1	74.7	92.3	82.7
S2	80.6	82.1	81.4
S3	87.5	92.3	89.2
S4	87.2	88.8	86.9
S5	92.1	75.3	82.8
S6	92.1	96.4	94.0
S7	90.6	84.2	87.0
S8	75.3	79.8	77.4
Mean ± STD	85.0 ± 6.3	86.4 ± 6.4	85.2 ± 4.6

### Online manipulation performance in grasping process

**Figure 8:**
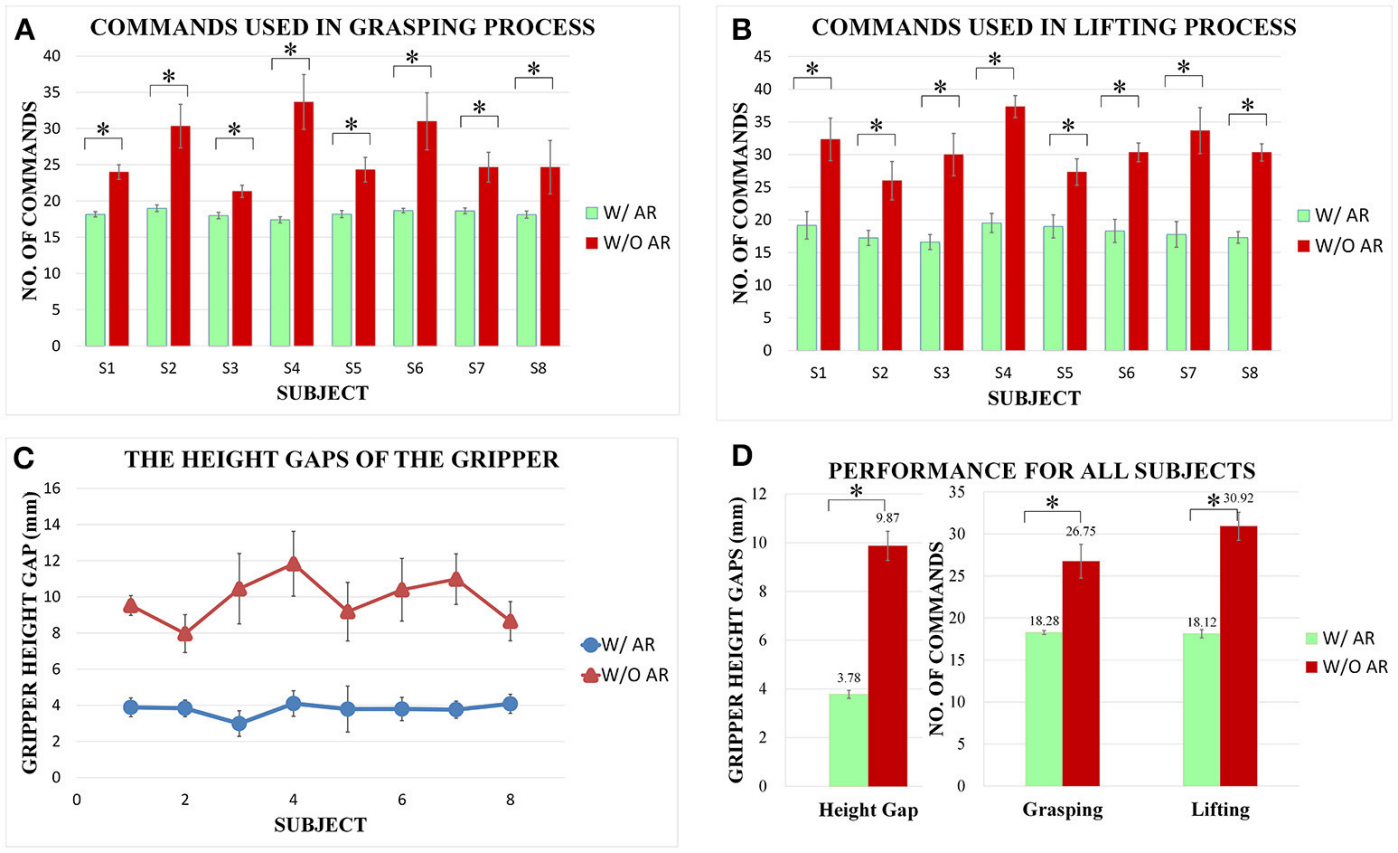
Comparisons of the number of trigger commands and the height gaps in the objects manipulation tasks between the system with AR feedback and those with visual inspection only. The statistically significant performance difference has been marked by “^*^” (*p* < 0.05). **(A)** The number of trigger commands used in the grasping process for each subject. **(B)** The number of trigger commands used in the lifting process for each subject. **(C)** The height gaps of gripper for each subject in the object lifting process. **(D)** The height gaps of the gripper and the number of trigger commands used in the grasping and lifting processes averaged over all the subjects.

The average number of commands used in the grasping process for each subject is shown in Figure 8A. The number of trigger commands used for the objects grasping with AR feedback is generally less than that with visual inspection only. In particular, for subject 4, the number of trigger commands has been reduced from 33 to 17 when the enhanced visual feedback is provided. With normal visual inspection only, (i.e., no AR feedback is provided), it is hard for the users to clearly observe the status of the grasping process, especially when the robotic arm hinders the objects from the subjects' view (e.g., Figure 7J). Furthermore, in order to grasp the object tightly, the user has to generate more controlling commands by the hybrid Gaze-BMI in the grasping process without AR feedback than that with AR feedback. By contrast, the aperture of the gripper and the simulated grasping force between the gripper and the objects are shown for the user with AR feedback in real time. Therefore, it is much easier for the user to handle the grasping process. The results have revealed that the grasping task can be completed with less trigger commands and more consistent performance across the subjects with AR feedback than that with visual inspection only. The number of trigger commands used in the grasping task with the AR feedback is statistically less than that without the AR feedback for each subject (ps1 = 0027, ps2 = 0.0022, ps3 = 0.0089, ps4 = 0.0032, ps5 = 0.0025, ps6 = 0.0018, ps7 = 0.0029, ps8 = 0.0010).

### Online manipulation performance in lifting process

The average number of commands used in the lifting process is shown in Figure 8B. In order to avoid the obstacle in the middle of the workspace, the user should control the gripper moving in the vertical direction until the height of the gripper is higher than the obstacle for a safe delivering. The number of commands generated from BMI has been reduced significantly with AR feedback. When no AR feedback is provided, it is hard for the user to decide whether the height of the robotic gripper is already higher than that of the obstacle in the lifting process. Therefore, to ensure a safe delivering, the user tends to generate more controlling commands by the hybrid Gaze-BMI. In the approach with AR feedback, a virtual box, whose height is equal to the altitude of the robotic gripper obtained from the Dobot engine, was presented right in the middle of the virtual obstacle. Furthermore, the height of the virtual box changes along with the altitude of the gripper in real time. In this way, the user can better perceive the status of the lifting process based on the enhanced visual feedback. The results have revealed that all the subjects can finish the lifting task in around 20 trigger commands with AR feedback. By contrast, much more commands are used in the same task with visual inspection only than the one with AR feedback. The number of trigger commands used in the lifting task with the AR feedback is also statistically less than that without the AR feedback for each subject (ps1 = 0.0054, ps2 = 0.0066, ps3 = 0.0089, ps4 = 0.0039, ps5 = 0.0135, ps6 = 0.0018, ps7 = 0.0036, ps8 = 0.0010).

The height gap of the robotic gripper for each user is shown in Figure 8C. The height gaps with AR feedback are generally smaller than those with visual inspection only for all subjects, which shows that the subject is capable of find out when to finish the lifting process in time with less efforts based on the enhanced visual feedback. Moreover, the results also show that the height gaps in the lifting task are much more consistent across the subjects with AR feedback than those without AR feedback. The gripper height gaps obtained with AR feedback are statistically smaller than those without AR feedback (ps1 = 0.0040, ps2 = 0.0066, ps3 = 0.0018, ps4 = 0.0040, ps5 = 0.0282, ps6 = 0.0018, ps7 = 0.0015, ps8 = 0.0021).

### Overall manipulation performance for all subjects

Figure 8D shows the average height gaps of the gripper as well as the average number of trigger commands used in the grasping and lifting processes for all the subjects with the system with or without AR feedback. The average height gap of the gripper is <4 mm with AR feedback, whereas it is more than 9 mm when only the visual inspection is provided, leading to a reduction in more than 50%. The average number of commands for all subjects decreases from 26.75 to 18.28 and 30.92 to 18.12 in the grasping and lifting processes, respectively. Furthermore, the standard deviation of the number of commands with AR feedback is smaller than that without AR feedback. This is because different subjects may have different understandings of the current task status with visual inspection only. By contrast, it is easier for all the subjects to perceive the task status with AR feedback, and to take advantage of the feedback information provided by AR interface in completing the grasping and lifting tasks. Therefore, the performance with AR feedback of all the subjects is more consistent than that with visual inspection only, indicating that the AR feedback indeed can enhance the performance of the hybrid Gaze-BMI controlled grasping and lifting processes in the objects manipulation tasks.

## Discussion

### Subject variability of the manipulation performance

Firstly, we will illustrate the necessity to remove the subject variability effect of the BMI decoding when evaluating the manipulation performance for the systems with or without the AR feedback. It is well-known that there is the BMI decoding performance variability across the subjects (Huster et al., 2015; Ouyang et al., 2017), which is also the case for our implementation of BMI (see subsection The Classification Performance of the BMI). Because the aim of our online experiments is to testify the possible manipulation performance improvement by introducing the AR feedback to the hybrid Gaze-BMI based robotic arm control system, the subject variability factor associated with the BMI decoding should be removed. To this end, the number of trigger commands used in both the grasping and the lifting process, and the height gaps of the robotic gripper in the lifting process were utilized as the indices for the system manipulation performance, since the commands only can be triggered when the motor imagery state has been detected successfully.

Secondly, the subject variability on the manipulation performance of the complete system will be discussed. As can be observed from Figure 8, these three manipulation performance indices are almost consistent across subjects when the AR feedback is provided in the system, whereas this is not the case for the system without the AR feedback. This is mainly due to the reason that the AR feedback can provide the timely hints for the user to switch on the next action. For example, once the subject observes the arrows overlaid over the gripper, which simulate the grasping force between the gripper and the object, the subject can stop generating the trigger commands by the hybrid Gaze-BMI. By contrast, when there is no AR feedback provided, the user has to rely on their own perception of the grasping status by normal visual inspection only. As a result, the manipulation performance of the system without the AR feedback has demonstrated significant subject variability.

### AR feedback vs. visual inspection only

The objects manipulation tasks with AR feedback and with visual inspection only are performed by the subjects, respectively. In this work, AR feedback is presented in the real scene, which will help the user to understand the meaning of the feedback information. The most significant advantage of AR feedback is that it can provide abundant and flexible information for the patients in an intuitive way via the visual communication channel. In specific, the change in color of the virtual rectangle surrounding the objects indicates the user's conformation of the selected objects, the width of a virtual box is used to represent the aperture of the gripper, the arrow stands for the simulated grasping force in the contacted phase, and the virtual box, whose length is the same as the altitude of the gripper, is overlaid right in the middle of the virtual wall. For the object manipulation tasks, the grasping and lifting processes are executed manually by the hybrid Gaze-BMI users with AR feedback. The hybrid Gaze-BMI can provide a sufficient degree of flexibility for the robotic arm control with the combined gaze selection and BMI control strategy. Meanwhile the subject can utilize the enriched visual information provided by the AR interface to establish the closed-loop control. The performance of the hybrid Gaze-BMI based system using AR feedback is improved notably compared to the one without AR feedback, in terms of both the number of commands used in the controlling process and the height gap of the robotic gripper.

It is necessary to point out that the AR feedback is not a rigid requirement in the objects manipulation tasks according to our experimental results, because the subject can also complete the tasks without AR feedback. However, the performance of the proposed method is improved significantly with the enhanced visual feedback. When no AR feedback is provided, the BMI users tend to rely exclusively on the visual feedback. However, the object may be hided from the field of view by the robotic gripper, in addition, it is hard to estimate the difference between the altitudes of the gripper and the height of the obstacle with the normal visual inspection only. Therefore, this approach may contribute to time-consuming and ineffective performance, thus increasing the workload on the BMI user. The experimental results has demonstrated that the closed-loop control for the grasping and lifting tasks can be achieved by the hybrid Gaze-BMI based system integrating with the AR guiding assistance. Furthermore, the performance of the BMI user with the enhanced visual feedback is improved significantly over that with visual inspection only.

### Fully automatic control vs. manual control

Previous studies have demonstrated that subjects can perform the objects manipulation tasks using the BMI. The object is selected in the workspace using gaze tracking (McMullen et al., 2014) or using EEG P300-evoked response to the visual cue over the object (Lenhardt and Ritter, 2010; Ying et al., 2017). In those studies, once the object is confirmed by the BMI user, the task will be completed by the robotic arm automatically without the user's intervention, which may fail to improve the user's level of gratification (Kim et al., 2012; Downey et al., 2016). Rather than completing the task automatically, we divide the task into five phrases. For the grasping and lifting tasks requiring fine operations where the human supervisory is desired, they are controlled by the BMI users manually. For the less demanding tasks, such as reaching, delivering and releasing, are completed by the robotic arm automatically once the movement intention is detected from the EEG signals.

The main challenge of the manual control is that the feedback information from the visual inspection only is not sufficient for the user, which may lead to time-consuming and ineffective grasping and lifting tasks (Johansson and Flanagan, 2009).

In order to achieve an effective and efficient manual control in the grasping and delivering processes, AR feedback is used to provide the user with the enhanced visual feedback information about the current status of the tasks. Specifically, the aperture and the altitude of the gripper are controlled manually by the user, and the user can decide when to stop the current action and switch to the next action by means of the information providing by AR interface. In this way, the user is able to maintain as much control as possible in the grasping and lifting processes via the hybrid Gaze-BMI, while obtaining the feedback information via the AR interface.

### Comparison with other BMI systems

It is important for patients working with assistive devices to restore their ability for performing activities of daily living such as objects manipulation. Patients with severs motor disabilities cannot fully benefit from assistive devices because of their limited access to the latest assistive products (Millan et al., 2010). To solve the problem, many researchers have focused on BMI based on both invasive and non-invasive neural signals (Nicolas-Alonso and Gomez-Gil, 2012; Chaudhary et al., 2016).

For the invasive BMI, the neural activities of the brain are measured using the electrodes placed on the surface of the cerebral cortex or implanted directly into the gray matter of the brain. Then the acquired neural signals are used to control the robotic arm continuously in three dimensional (Hochberg et al., 2012; Collinger et al., 2013; Downey et al., 2016). In Hochberg et al. (2012), the neural activity is collected with the implanted microelectrode array, and the endpoint velocity of the robotic arm is continuously mapped from the decoded neural activity without other assistance. However, it is very difficult to establish a fine continuous mapping for the low-level control of the robotic arm from the noisy neural activities, two tetraplegia and anarthric patients can only complete the tasks in about 60% trials. Moreover, it has to implant the electrodes via surgical procedures with medical risks.

For non-invasive BMI, various modalities have been proposed such as fMRI, fNIRS, MEG, and EEG (Nicolas-Alonso and Gomez-Gil, 2012). Although fMRI and MEG have better spatial resolution compared with EEG, these two methods need expensive equipment which is non-portable (Muthukumaraswamy, 2013). fNIRS is a relative new measurement method which employs infrared light to characterize non-invasively acquired fluctuations in cerebral metabolism during neural activity. Though fNIRS uses low cost equipment and an acceptable temporal resolution, one of the major limitations of fNIRS based BMI is the inherent delay of the dynamic response (Naseer and Hong, 2015). Therefore, the EEG signals by placing the electrodes on the surface of the scalp are mostly studied, due to its high temporal resolution, few risks to the user and requires less expensive equipment.

It has been shown that the EEG signals acquired during multiple types of motor imagery tasks can be decoded for moving the robotic arm in multiple directions (Wang et al., 2012). Nevertheless, it is difficult to achieve an accurate classification of multiple mental states using EEG signals of poor signal-to-noise ratio. Furthermore, it is a challenging task in practice for the BMI user to switch among multiple mental states constantly. It is much easier to implement a 2-class based BMI, but it lacks sufficient flexibilities for controlling the robotic arm. Therefore, the hybrid Gaze-BMI is used in our study: the user's gaze points on the monitor are provided by the eye-tracking for the object selection, and the movement intention of the user can be detected by the BMI for confirming the selected object or initiating the control command to be executed on the selected object.

### Limitations and future work

One of the drawbacks of our study is that AR feedback is provided to the subjects via the computer monitor. It will reduce the hommization of this system and limit the scope of application to communication with the assistive devices via the computer monitor. Besides, we are also aware that patients may interact with various objects with different size and colors in activities of daily living, while the object manipulated in this study are of the same size. Besides, the AR feedback in our paper is based on the difference between the width of the objects and the gripper aperture, which may limit the usability of this method in activities of daily living.

The purpose of our study is to find out whether the hybrid Gaze-BMI user can benefit from AR feedback to perform the closed-loop control in the grasping and lifting tasks. Such a functional ability will be enhanced with the following improvements in our future work. Firstly, the ponderous computer monitor can be replaced by the wearable AR glasses integrated with eye tracking to increase the flexibilities and the scope of application. Secondly, the gripper with pressure sensors will be used to monitor the grasping status, and the real force generated in the contacting phrase will be presented to the user using AR techniques. Thirdly, the participants in this study are all healthy individuals, the feasibility of this method will be evaluated on the patients with motor impairments after stroke. Lastly, the performance of the proposed system will be integrated with other kinds of feedback interfaces, such as the haptic feedback, the auditory feedback, and so on.

In addition, the hybrid Gaze-BMI and the proposed AR feedback method for the assistive robot used in our paper can be seamlessly applied for the rehabilitation robot. For example, patients use eye gaze to indicate a desired position in a real environment setting, the robotic arm exoskeleton can be used to assist the patients to perform the reaching movement along online human-like generated trajectories when the self-initiation movement intention is detected with BMI. Besides, the wearable AR glasses can be exploited for the user to provide AR feedback for the operation status in order to implement an effective closed-loop control.

## Conclusion

In this paper, we have proposed a novel AR guiding assistance for closing the hybrid Gaze-BMI based robotic arm control loop. The subjects are trained to reach, grasp, lift, deliver and release an object while avoiding the obstacle in the workspace, by operating a robotic arm with the hybrid Gaze-BMI. Instead of perceiving the current states of the tasks by the visual inspection only, the AR interface has been established in the real scene from the workspace to feedback the current gripper status for the subjects. The hybrid Gaze-BMI users are instructed to rely on the AR feedback information while accomplishing the objects manipulation tasks.

The experimental evaluation of the complete setup was conducted with eight healthy subjects. The average BMI classification accuracy across the subjects is 85.16 ± 4.83%. The number of trigger commands used for controlling the robotic arm to grasp and lift objects with AR feedback has reduced significantly compared to that without AR feedback, and the height gaps of the gripper in the lifting process have decreased more than 50% compared to those trials with normal visual inspection only. The results have revealed that the hybrid Gaze-BMI user can benefit from the information provided by the proposed AR interface, improving the efficiency and reducing the cognition load during the hybrid Gaze-BMI controlled grasping and lifting processes.

## Author contributions

HZ and YW designed the study, analyzed the data and wrote the manuscript. CW and PJ set up the experiment platform, BX and LZ performed the experiment. AS, JL, HL, and PW were involved in critical revision of the manuscript. All authors read and approved the final manuscript.

### Conflict of interest statement

The authors declare that the research was conducted in the absence of any commercial or financial relationships that could be construed as a potential conflict of interest.
